# UNUSUAL PRESENTATION OF GENERALIZED MACULAR AMYLOIDOSIS IN A YOUNG ADULT

**DOI:** 10.4103/0019-5154.44802

**Published:** 2008

**Authors:** Mohan H Kudur, Sathish Pai B, Sripathi H, Smitha Prabhu

**Affiliations:** *From the Department of Dermatology, Kasturba Medical College, Manipal, India*

**Keywords:** *Generalized macular amyloidosis*, *poikilodermatous appearance*, *congo red stain*

## Abstract

Macular amyloidosis is a common problem seen dermatology out-patient department. Generalized macular amyloidosis presenting with a poikilodermatous appearance is rare. In our case, an 18-year-old male presented with generalized hypopigmented macules with a poikilodermatous appearance of 10-year duration. His developmental milestones were normal with negative family history of similar complaints. Histopathology of hyperpigmented lesions revealed hyperkeratosis and acanthosis of epidermis and hypopigmented lesion showing only hyperkeratosis. Both lesions were showing the deposition of amorphous, hazy material in the tips of papillary dermis with perivascular inflammatory infiltrate. Congo red staining of the amorphous material was positive for amyloid.

## Introduction

Macular amyloidosis is the most subtle of the cutaneous amyloidosis. The original description of this entity was made by Palitz and Peck in 1952.^1^ Poikilodermatous appearance in macular amyloidosis, though described in standard textbooks, is rarely seen. Typically, macular amyloidosis presents as “rippled” pattern of pigmentation over upper back, extensor aspect of arms and forearms and legs. Histopathology shows deposition of amorphous, hazy amyloid in the tips of the papillary dermis. Amyloid shows green birefringence when stained with Congo red and viewed under polarized microscopy. Direct immunofluorescence shows Ig G, Ig M and C3 deposition in amyloid.

## Case Report

**Fig. 1:**
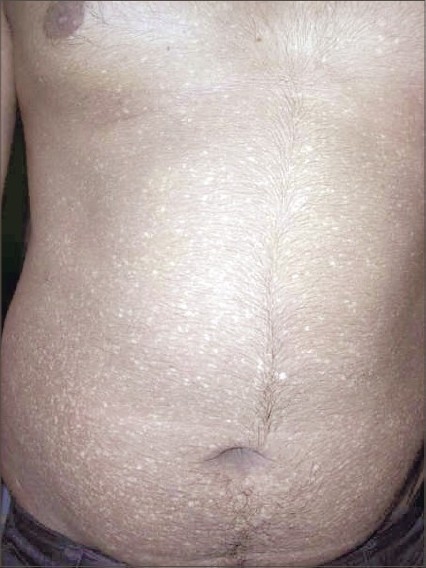
Hypopigmented macules over abdomen

**Fig. 2:**
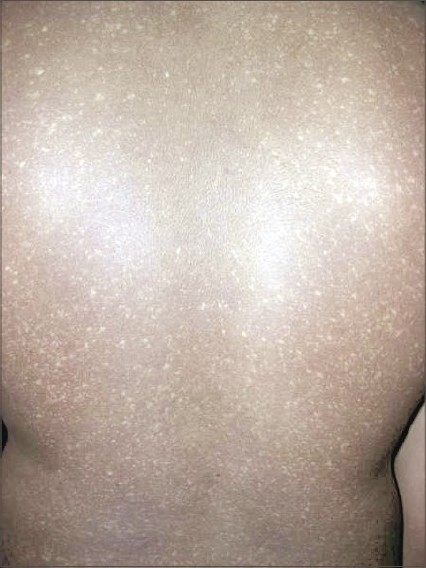
Hypopigmented and hyperpigmented macules over back

**Fig. 3:**
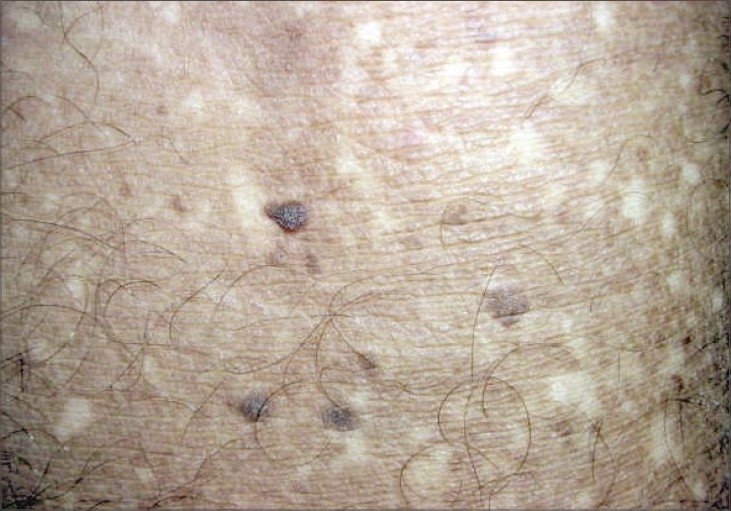
Poikilodermatous appearance on closer view

**Fig. 4:**
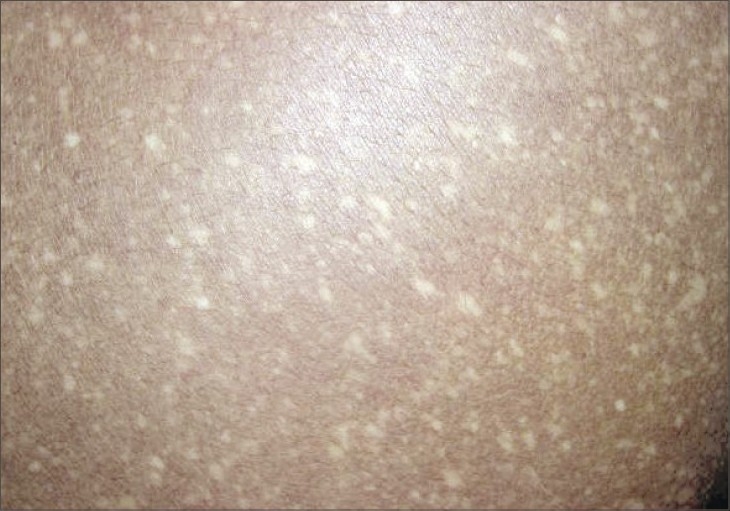
Hypopigmented macules over the lower back

A 18-year-old male presented to the skin out-patient department with asymptomatic, generalized hypopigmented macules with a poikilodermatous appearance of 10-year duration. It had started over abdomen and then gradually spread to involve back, upper and lower limbs. No history of oral, genital, hair or nail lesions. His developmental milestones were normal. There was no history of any other skin lesions in the past. There was no history of photosensitivity and similar complaints in the family, and no history of rubbing of skin with brush or any material while taking bath. On examination, hypopigmented macules varying from 4 to 6 mm in diameter are observed involving the abdomen, back, upper limbs and lower limbs (Figs. 1–4). Oral mucosa, genitalia, palms and soles was spared. Skin biopsy was done from both the hyperpigmented and hypopigmented macules.

**Fig. 5:**
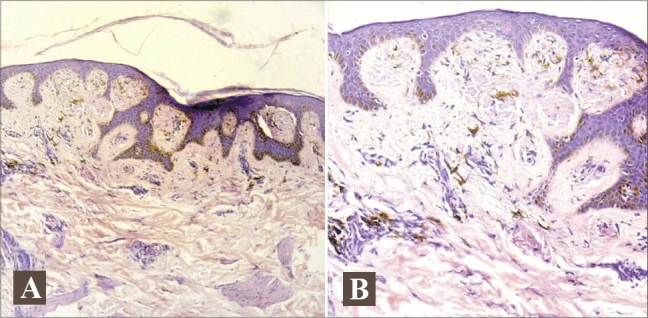
(A) Histopathology of a hyperpigmented macule showing hyperkeratosis; deposition of amorphous material is observed at the tips of papillary dermis. (B) Histopathology from a hypopigmented macule showing amorphous, hazy deposition in the papillary dermis.

**Fig. 6:**
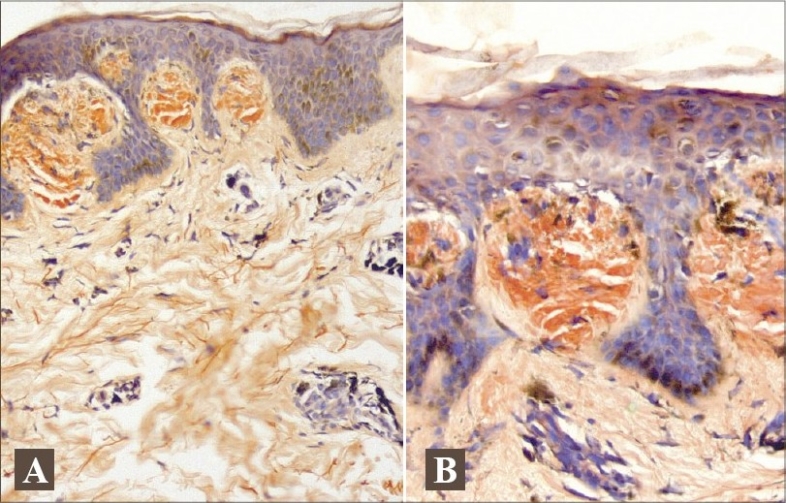
(A) Congo Red staining of Amyloid at tips of papillary dermis. (B) Closer view

Histopathology from the hyperpigmented macule revealed epidermis showing hyperkeratosis and acanthosis. Deposition of a hazy material was noticed in the papillary dermis with perivascular inflammatory infiltrate involving the upper dermis (Figs. 5 and 6). Hypopigmented macule also showed deposition of hazy, amorphous material in the papillary dermis. Both specimens were positive for amyloid with Congo red stain. Complete blood counts, urine routine, fasting and post-prandial blood sugar, liver function and renal function tests were normal. Thyroid stimulating hormones, T3 and T4, were within the normal limits.

## Discussion

It is a form of primary localized cutaneous amyloidosis (PLCA). Congo red staining was positive for amyloid deposits in this patient. The main feature of primary cutaneous amyloidosis is the accumulation of amyloid in previous apparently normal skin without any deposits in the internal organs.

It is more common among central and south Americans, middle east population and Asians who have dark skin. It is rare among European and north-American races. This apparent racial differentiation, in the case of Japanese, may be partially contributed by the habit of rubbing the skin vigorously with nylon towel or brush (“friction amyloidosis”).^2^,^3^ There is a postulated association of primary localized cutaneous amyloidosis with notalgia paresthetica.^4^

The importance of genetic factors in the development of PLCA is emphasized by the occurrence of familial cases.^5^

The lesions consist of macular hyperpigmented areas predominantly on the upper back (interscapular area), buttocks, chest (over clavicles, on the ribs), breast and extremities (shins and forearms). Lesions appear as grayish brown macules, 2–3 mm in diameter. A reticulate or “rippled” pattern of pigmentation is a characteristic diagnostic feature in many cases of macular amyloidosis. Macules may have mild to moderate pruritus. Sometimes pruritus may be absent. Unusual variants of PLCA include macular forms with diffuse hyperpigmentation,^6^ simulating nevoid hyperpigmentation^7^ and a poikilodermatous form.^8^ Amyloidosis cutis dyschromica is assumed to be a congenital disorder with hypersensitivity to UVB radiation, with possible DNA repair defects; hyperpigmented and hypopigmented xerotic lesions with deposits of amyloid in papillary dermis occur in sun-exposed skin.^9^ The condition usually presents in early adult life and persists for many years. Both sexes may be equally affected. Macular and lichen amyloidosis frequently coexist, giving evidence to the concept of biphasic amyloidosis.

Histopathology shows amyloid deposited in and confined to the papillary dermis and does not extend beyond the subpapillary plexus. Early lesions contain small multifaceted amorphous globules within the papillae, which are easily missed without use of special stains. Later lesions show globules, which coalesce, expand the papillae, and displace the rete ridges laterally. Special stains for amyloid include the triphenyl methane dyes methyl and cresyl violet for the demonstration of metachromasia, the periodic acid schiff's method, the cotton dyes Congo red and Sirius red with or without fluorescence or polarized light and fluorescence with thiazole dyes such as thioflavine T. Alternative cotton dyes including Pagoda red, RIT scarlet no. 5 and RIT cardinal no. 9 may be used. Fluorescence methods using an optical brightener for cellulose and immunohistochemical staining with anti-SAP have also been advocated. False-positive staining with Thioflavine T is observed with stromal hyaline deposits, collagen fibers and colloid bodies in lichen planus. Amyloid exhibits green birefringence with Congo red when viewed under polarizing microscope. This is because of perpendicular arrangement of fibrillary deposits. AL-type amyloid, unlike AA-type amyloid, retains its affinity for Congo red and its typical polarization characteristics after exposure to potassium permanganate.^10^

Direct immunofluorescence usually reveals focal Ig G, Ig M and C3 in the amyloid. To conclude, amyloidosis can present with various pigmentary abnormalities such as hypopigmentation, poikilodermatous appearance and nevoid appearance other than the typical “rippled pattern” of hyperpigmentation.

## References

[1] Brownstein MH, Hashimoto K (1072). Macular Amyloidosis. Arch Dermatol.

[2] Hashimoto K, Ito K, Kumakiri M, Headington J (1987). Nylon brush macular amyloidosis. Arch Dermatol.

[3] Onuma L, Vega M, Arenas R, Dominguez L (1994). Friction amyloidosis. Int J Dermatol.

[4] Peña-Penabad MC, Garcia-Silva J, Armijo M (1995). Notalgia paresthetica and Macular amyloidosis: Cause – effect relationship?. Clin Exp Dermatol.

[5] De Pietro WP (1981). Primary familial cutaneous amyloidosis: A study of HLA antigens in a puerto Rican family. Arch Dermatol.

[6] Wong CK, Lee JY (1996). Macular amyloidosis with widespread diffuse pigmentation. Br J Dermatol.

[7] Black MM, Maiback HI (1974). Macular Amyloidosis simulating naevoid hyperpigmentation. Br J Dermatol.

[8] Serna-Perez MJ, Vázquez-Doval FJ, Idoate M, Sola Casas MA, Quintanilla E (1992). Extensive macular amyloidosis associated with poikiloderma. Int J Dermatol.

[9] Moriwaki S, Nishigori C, Horiguchi Y, Imamura S, Toda K, Takebe H (1992). Amyloidosis cutis dyschromica: DNA repair reduction in the cellular response to UV light. Arch Dermatol.

[10] Wright JR, Calkins E, Humphrey RL (1977). Potassium permanganate reaction in amyloidosis: A histologic method to assist in differentiating forms of this disease. Lab Invest.

